# Positive mood enhances accessibility of unrelated concepts in the first language but not in the foreign language

**DOI:** 10.1371/journal.pone.0353990

**Published:** 2026-08-12

**Authors:** Piotr Żukowski, Marcin Naranowicz

**Affiliations:** 1 Laboratory for Social Neuroscience, Faculty of English, Adam Mickiewicz University, Poznań, Poland; 2 Cognitive Neuroscience Center, Adam Mickiewicz University, Poznań, Poland; Education University of Hong Kong, HONG KONG

## Abstract

Prior research has shown that, compared to a negative mood, a positive mood facilitates spreading activation within semantic memory in the first language. Yet, little is still known about neurocognitive mechanisms underlying mood effects on semantic processes in the foreign language. Here, we show that a positive mood enhances accessibility of unrelated concepts within semantic memory in the first but not the foreign language. Highly proficient Polish–English bilingual women were induced into positive and negative mood states with animated films and made semantic relatedness judgements about closely related, weakly related, and unrelated prime–target pairs in their first and foreign languages. Mean N400 responses were analysed as neural markers of spreading activation within semantic memory. Our results revealed reduced N400 responses to unrelated word pairs under a positive compared to a negative mood only in the first language. Critically, no mood-driven effects emerged for the foreign language. These findings provide novel evidence that a positive mood broadens spreading activation within semantic memory only in the first language context, making distant concepts more accessible. Critically, they also highlight mood-independent responding when bilinguals operate in their foreign language.

## 1. Introduction

Mood incessantly filters through our daily thoughts, decisions, and actions, significantly influencing our understanding of reality [1]. Given its pervasive nature and its role in shaping cognitive processes, mood also impacts everyday communication by affecting how individuals express themselves and interpret utterances from others. This strongly suggests that mood is not merely a background factor but an indispensable component of language comprehension, which may also dynamically modulate the mechanisms underlying spreading activation within semantic memory.

As bilingual and multilingual individuals constitute more than half of the world’s population [2], for most people, switching between their first (L1) and foreign (L2) language is a routine experience. Understanding how mood influences bilingual interactions is therefore critical for improving communication in globalised workplaces, language learning environments, and mental health contexts. However, despite its practical relevance, research on the influence of mood on bilingual language processing is still at a relatively early stage of development (see [3] for a review). This leaves a significant gap in research which could inform strategies for enhancing clarity and reducing misunderstandings in multilingual settings.

### 1.1. Semantic processing

The processing of semantic information has often been described in terms of spreading activation mechanisms within semantic network models [4,5]. Such frameworks conceptualise semantic memory as a hierarchical network of conceptual nodes, while the links that connect them reflect the strength of associative or semantic relatedness. In such models, concepts that are more strongly related are positioned closer in the network than those that share weaker associations [4]. Activation of one concept spreads through links to neighbouring nodes, temporarily lowering the threshold necessary for their activation, which results in enhanced retrieval of closely related concepts [4]. Therefore, spreading activation has been extensively used to explain the facilitatory and inhibitory effects observed in semantic priming experiments [6–8].

Semantic priming has become one of the fundamental experimental paradigms in cognitive psychology (see [9,10] for reviews). It has been defined as a facilitation in the processing of a lexical or visual stimulus (i.e., the target) when it is presented after a semantically related stimulus (i.e., the prime; e.g., *dog–cat*), compared to when the prime–target pairs are unrelated (e.g., *table–cat*) [9,11]. At a behavioural level, semantic priming manifests as shorter response times and improved accuracy for related compared to unrelated prime–target pairs [12–14]. At an electrophysiological level, semantic priming is often reflected in the N400 amplitude changes [15,16]. The N400 is a negative-going event-related potential (ERP) component peaking in amplitude around 300–500 ms post stimulus onset over centro-parietal electrode sites, commonly interpreted as a neural marker of lexico-semantic processing [17]. Monolingual research has consistently shown that semantically related compared to unrelated prime–target pairs elicit reduced N400 responses [18–20]. For instance, Rataj et al. [21] used prime–target word pairs of three different degrees of relatedness: closely related (e.g., *gum–throat*), weakly related (e.g., *ribs–throat*), and unrelated pairs (e.g., *grass–throat*). They found a graded N400 pattern with the largest amplitudes for unrelated, intermediate for weakly related, and smallest for closely related word pairs. These results suggest that lexico-semantic processes are susceptible to the degree of semantic relatedness.

Semantic priming has been widely applied in bilingual research (see [22] for a review). For instance, Kotz [23] integrated the semantic priming paradigm with a lexical decision task in an ERP study involving highly proficient Spanish–English (L1–L2) bilinguals. They compared semantic priming effects in the N400 time window focusing on semantic associations (related [e.g., *girl–boy*] vs. unrelated [e.g., *house–boy*]) and semantic categories (related [e.g., *junior–boy*] vs. unrelated [e.g., *pond–boy*]). They observed larger N400 responses to unrelated compared to related pairs for both semantic associations and categories, regardless of language of operation. Such patterns indicate that similar lexico-semantic processes may be activated by different semantic relations in L1 and L2 in highly proficient bilinguals.

### 1.2. Mood effects on semantic processing

Mood has been defined as a background low-intensity affective state that fluctuates over time from feeling positive to negative [1]. The *Affect-as-Information* hypothesis [24,25] proposes that mood states, whether positive or negative, shape cognitive processing by eliciting context-sensitive patterns of thought. Within this socio-cognitive framework, a positive mood is typically associated with heuristic, assimilative, and relational thinking. It promotes reliance on prior knowledge, a broad attentional scope, cognitive fluency, and a tendency to explore. In contrast, a negative mood supports accommodative and referential processing, characterised by a narrowed attentional focus, increased cognitive effort, and a tendency to exploit available information (see [1] for a review).

Monolingual research has shown that positive and negative moods differently shape semantic processes (see [3,26] for reviews). Behavioural studies have concentrated on semantic memory organisation and indicated that, while a negative mood may generally decrease spreading activation within semantic memory [27–29], a positive mood may facilitate activation of close [30] and/or remote associates [27]. Such patterns have been linked to increased breadth of attentional selection [31] and greater reliance on general knowledge [32] in a positive mood compared to a negative mood. Then, electrophysiological studies have mostly concentrated on lexico-semantic processes at a sentence level, showing that a positive mood may facilitate access to distantly related concepts belonging to the same [33] and/or different semantic categories [34,35], in contrast to a negative mood. This has been associated with the activation of heuristics-based and assimilative mechanisms under a positive mood and detail-oriented and accommodative mechanisms under a negative mood during lexico-semantic processing (see [3] for a review).

Bilingual research has extended these findings by showing that lexico-semantic processing in L2 compared to L1 might be less susceptible to mood fluctuations [36–38; but see 39,40]. For instance, Naranowicz et al. [36] explored mood effects on semantic violations in highly proficient L1-dominant Polish–English bilinguals. They reported increased N400 responses to words semantically incongruent compared to congruent with the sentence context. Critically, N400 responses to semantically incongruent words were reduced in L1 compared to L2 in a positive mood, with no such between-language differences in a negative mood. While such patterns point to a facilitatory effect of a positive mood on semantic violation processing in L1 relative to L2, the relationship between mood-driven neural dynamics and the processing of semantic versus associative links in L2 remains largely unexplored.

### 1.3. Present study

This electrophysiological study aims to uncover the neural mechanisms underlying the relationship between mood and semantic processing in L1 and L2, with particular attention to the role of semantic relatedness. To this end, 32 highly proficient L1-dominant Polish–English bilinguals (all women) were induced into positive and negative moods with emotionally evocative animated films. Following mood induction, participants made semantic relatedness judgements about three types of prime–target pairs, both in L1 and L2: closely related pairs (e.g., *interpreter–translator*), weakly related pairs (e.g., *researcher–translator*), and unrelated pairs (e.g., *brake–translator*). Critically, unlike in prior electrophysiological studies that concentrated on lexico-semantic processing at a sentence level [33,34,36], we employed the semantic priming paradigm with word pairs to directly test how mood shapes the architecture of semantic memory in L1 and L2. This approach allowed us to isolate activation patterns within semantic networks without the interpretive demands of sentence contexts, thereby providing a more direct assessment of how positive and negative moods modulate spreading activation among closely related, weakly related, and unrelated concepts in each language.

Building on previous research, we overall expected to observe qualitative mood-dependent differences between semantic mechanisms in L1 and L2. First, we expected to observe facilitated spreading activation mechanisms within semantic memory through a reduction in the activation threshold for distantly related concepts under a positive mood compared to a negative mood in L1 (Hypothesis 1). This would be reflected in reduced N400 responses for weakly related and/or unrelated prime–target pairs in L1 under a positive compared to a negative mood [33–35].

Second, we hypothesised a mood-independent effect on spreading activation within semantic memory in L2 (Hypothesis 2). This would be reflected in a graded N400 pattern irrespective of the mood type in L2, with the largest N400 responses for unrelated pairs, intermediate for weakly related pairs, and the smallest for closely related pairs in both mood conditions [36–38].

## 2. Materials and methods

### 2.1. Participants

The original sample included thirty-three participants, one of whom was excluded from the analysis due to excessive blinking. Consequently, thirty-two right-handed highly proficient L1-dominant Polish–English (L1–L2) bilinguals aged 20–30 (*M* = 24.13 years, 95% CI [23.39–24.86]) participated in the experiment proper. All participants were neurotypical and had normal or corrected-to-normal vision and hearing and no history of psychiatric, affective, or language-related disorders (see also Table 1). Participants were students and graduates of the Faculty of English, Adam Mickiewicz University, Poznań, Poland (AMU). Only women were invited to participate in the experiment proper, as the previous electrophysiological research has revealed gender-related variations in mood effects on language comprehension [35,38]. Each participant received a voucher worth 120 PLN as compensation.

**Table 1:** Participants’ characteristics (means with 95% confidence intervals in square brackets and ranges in round brackets).

Characteristic	Score	Characteristic	Score
Handedness^1^	87.81% [81.08, 94.54] (30.00–100.00)	L1 dominance^3^	54.88% [53.06, 56.68] (47.00–68.00)
Depression^2^	10.12% [7.11, 13.13] (.00–33.33)	L2 dominance^3^	54.91% [49.74, 58.08] (.00–68.00)
Anxiety^2^	5.51% [3.76, 7.26] (.00–19.04)	L3 dominance^3^	20.97% [15.48, 26.46] (.00–42.00)
Stress^2^	18.45% [13.72, 23.18] (.00–61.90)	L1 immersion^3^	93.72% [93.12, 94.32] (91.00–97.00)
L1 proficiency^3^	96.16% [94.01, 98.3] (79.00–100.00)	L2 immersion^3^	73.12% [69.89, 76.36] (39.00–92.00)
L2 proficiency^4^	92.39% [90.00, 94.79] (79.00–100.00)	L3 immersion^3^	26.41% [19.80, 33.01] (.00–57.00)
L2 proficiency ^3^	89.25% [86.92, 91.58] (79.00–100.00)	L2 age of acquisition^3^	6.44 years [5.62, 7.25] (1.00–14.00)
L3 proficiency^3^	38.09% [27.47, 48.72] (.00–79.00)	L3 age of acquisition^3^	18.22 years [16.86, 19.58] (10.00–25.00)

^1^Edinburgh Handedness Inventory [41] (translated into Polish for the purpose of the present project): left-handedness (−100– − 28), ambidexterity (−29–48), and right-handedness (48–100). ^2^DASS–21 [42] (translated into Polish by Makara-Studzińska et al.): normal (0–21%), mild (22–31%), moderate (32–48%), severe (49–64%), and extremely severe (65–100%) levels of depression, anxiety, and stress. ^3^Language History Questionnaire 3.0 [43] (translated into Polish by Naranowicz & Witczak): first language (L1): Polish; second language (L2): English; third languages (L3s): none, Chinese, French, German, Japanese, Norwegian, Spanish, and Russian. ^4^LexTALE [44].

### 2.2. Materials: Prime–target pairs

In total, 120 Polish (L1) and 120 English (L2) nouns were selected as target words. Each target was paired with three prime words to create unique pairs that varied in the semantic relatedness status: 120 closely related, 120 weakly related, and 120 unrelated word pairs in each language (*N*_*Total*_ = 720; see Table 2). All words were controlled for their levels of frequency, valence, arousal, concreteness, age of acquisition, and the number of letters across the three word pair types and the two languages (see S1 Appendix). Polish–English cognates, polysemous words, as well as interlingual homographs and homophones were not used as either primes or targets.

**Table 2:** Examples of experimental stimuli with their semantic relatedness ratings (means with 95% confidence intervals).

Selected Polish (L1) stimuli						
Closely related primes		Weakly related primes		Unrelated primes		Target
płot	6.05 [5.73, 6.37]	tama	3.95 [3.28, 4.62]	mydło	1.00 [1.00, 1.01]	brama
okno	5.60 [5.02, 6.17]	komin	4.50 [4.10, 4.91]	ziemia	1.54 [1.23, 1.85]	drzwi
sala	6.05 [4.61, 6.08]	łazienka	3.30 [2.57, 4.03]	pasza	1.00 [1.00, 1.01]	gabinet
język	6.25 [5.99, 6.51]	włosy	4.05 [3.33, 4.77]	obóz	1.01 [1.00, 1.02]	gardło
pokojówka	6.35 [6.08, 6.62]	opiekun	4.65 [4.39, 4.91]	krawędź	1.20 [1.01, 1.39]	lokaj
namiot	6.12 [5.82, 6.42]	gniazdo	4.40 [3.55, 5.25]	jamnik	1.01 [1.00, 1.02]	szałas
tarcza	6.35 [6.08, 6.62]	kamizelka	4.20 [3.43, 4.97]	odpływ	1.01 [1.00, 1.02]	zbroja
**Selected English (L2) stimuli**						
meeting	5.90 [5.23, 6.56]	summit	3.65 [2.80, 4.50]	plant	1.32 [1.10, 1.53]	assembly
latitude	5.73 [5.09, 6.37]	axis	4.21 [3.45, 4.98]	shutter	1.25 [1.07, 1.42]	equator
width	5.80 [5.24, 6.36]	span	3.60 [2.81, 4.39]	sandwich	1.20 [.96, 1.44]	height
harness	6.30 [5.99, 6.61]	scarf	3.55 [2.97, 4.13]	spleen	1.01 [1.00, 1.02]	leash
week	5.70 [4.91, 6.49]	holiday	4.45 [3.82, 5.08]	floor	1.00 [1.00, 1.01]	month
shovel	6.40 [6.08, 6.72]	spoon	4.40 [3.73, 5.07]	parrot	1.23 [.99, 1.47]	spade
interpreter	6.65 [6.38, 6.92]	researcher	4.15 [3.56, 4.74]	brake	1.01 [1.00, 1.01]	translator

Semantic relatedness scale: 1 – *very unrelated words / bardzo niepowiązane słowa*, 7 – *very related words / bardzo powiązane słowa*

**Fig 1:**
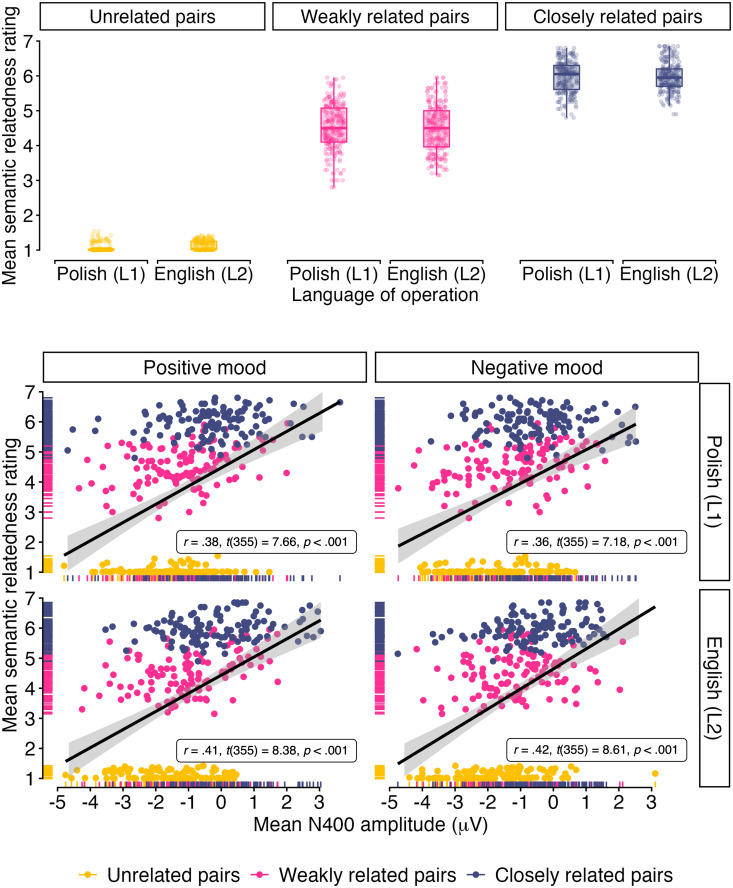
The top panel represents the distribution of mean semantic relatedness ratings in the norming study. The bottom panel represents the results of Pearson’s correlation analyses, showing moderate positive correlations between mean semantic relatedness ratings and mean N400 amplitude (i.e., an increase in semantic relatedness rating is accompanied by a decrease in N400 amplitude) across all conditions.

Each prime–target pair was rated by 20 highly proficient Polish–English bilinguals in terms of the degree of semantic relatedness on a 7-point scale in a separate norming study. The ratings validated the assignment of each pair to their respective category, with closely related pairs receiving the highest semantic relatedness scores (*M*_*L1*_ = 5.97, 95% CI [5.88, 6.05]; *M*_*L2*_ = 5.98, 95% CI [5.90, 6.05]), weakly related pairs receiving intermediate scores (*M*_*L1*_ = 4.53, 95% CI [4.41, 4.65]; *M*_*L2*_ = 4.50, 95% CI [4.37, 4.62]), and unrelated pairs receiving the lowest scores (*M*_*L1*_ = 1.05, 95% CI [1.04, 1.08]; *M*_*L2*_ = 1.12, 95% CI [1.10, 1.15]; see S1 Appendix and Fig 1).

Additionally, distributional semantic similarity between prime–target pairs was computed using cosine similarity derived from 300‑dimensional pretrained fastText word embeddings [45] for Polish and English. For each word, embedding vectors were retrieved using the *fasttext* package in R [46], and pairwise similarity was calculated as the cosine between the corresponding vectors. This measure ranges from −1–1, with higher values indicating greater distributional semantic relatedness. Mean similarity values were additionally computed across experimental conditions to verify that the manipulation produced systematic differences in relatedness.

Because distributional similarity is shaped by patterns of co-occurrence in natural language, it also captures aspects of lexical relationships related to associative structure. Accordingly, cosine similarity was treated as a complementary, corpus‑based, yet indirect estimate of associative strength, extending human semantic relatedness ratings by providing a continuous and scalable index of relationships between primes and targets [47–49].

**Fig 2:**
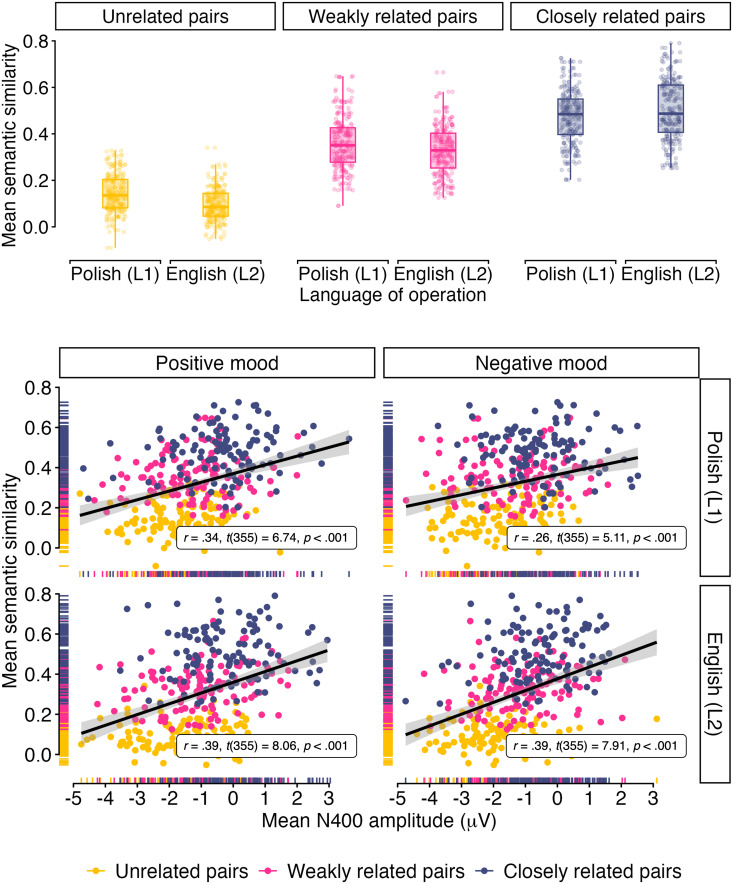
The top panel represents the distribution of cosine-based mean semantic similarity values. The bottom panel represents the results of Pearson’s correlation analyses, showing moderate positive correlations between mean semantic similarity values and mean N400 amplitude (i.e., an increase in mean semantic similarity is accompanied by a decrease in N400 amplitude) across all conditions.

Cosine similarity further validated the assignment of each pair to its respective category: closely related pairs showed the highest values (*M*_*L1*_ = .48, 95% CI [.45,  .50]; *M*_*L2*_ = .51, 95% CI [.49,  .54]), weakly related pairs showed intermediate values (*M*_*L1*_ = .36, 95% CI [.34,  .38]; *M*_*L2*_ = .33, 95% CI [.31,  .35]), and unrelated pairs showed the lowest values (*M*_*L1*_ = .14, 95% CI [.13,  .16]; *M*_*L2*_ = .10, 95% CI [.08,  .11]; see S2 Appendix and Fig 2). This pattern may also point to a likely graded variation in associative strength across pair types.

### 2.3. Materials: Mood-inducing films

To induce the targeted positive and negative mood states, participants were exposed to 8 positive and 8 negative mood-inducing animated films in two separate blocks. They were 90-second, non-narrative, physiologically stimulating, and emotionally potent films, adapted from a study by Naranowicz et al. [38], where each film was evaluated on 7-point valence and physiological arousal scales. On average, the positive mood-inducing films were rated as more positive and comparably arousing relative to the negative mood-inducing films (see Table 3). Each of the 16 selected films was divided into two 45-second fragments, providing a total of 32 film fragments presented in both mood conditions (i.e., 24 min).

**Table 3:** Mood-inducing films’ characteristics (means with 95% confidence intervals in square brackets and ranges in round brackets) adapted from Naranowicz et al. [38].

Characteristic	Positive mood-inducing films	Negative mood-inducing films	Film type comparisons*
Valence ratings	5.08 [4.93, 5.23] (1–7)	2.23 [2.05, 2.41] (1–7)	*t*(390.18) = −23.81, *p* < .001
Arousal ratings	3.74 [3.45, 4.02] (1–7)	3.81 [3.55, 4.07] (1–7)	*t*(396.36) = .36, *p* = .720

*based on Welch two-sample t-test.

### 2.4. Procedure

All experimental procedures were approved by the Ethics Committee for Research Involving Human Participants (AMU; Resolution No. 34/2019/2020). Prior to data collection, all participants were screened online using DASS-21 [42] and LexTALE [44]. Before the experimental procedure started onsite, all participants provided their written informed consent. The experiment proper was conducted at the Psychophysiology of Language and Affect Laboratory, AMU. During EEG cap preparation, participants completed Edinburgh Handedness Questionnaire [41] and Language History Questionnaire [43].

Within one experimental session, participants completed both mood blocks in a counterbalanced order, each involving the presentation of either 16 positive or 16 negative affectively evocative film fragments. To enhance the effectiveness of mood induction, participants were instructed to put themselves in the targeted mood [50] and imagine themselves as one of the protagonists [51]. Participants were asked to rate their current emotional state before and after each mood block.

**Fig 3:**
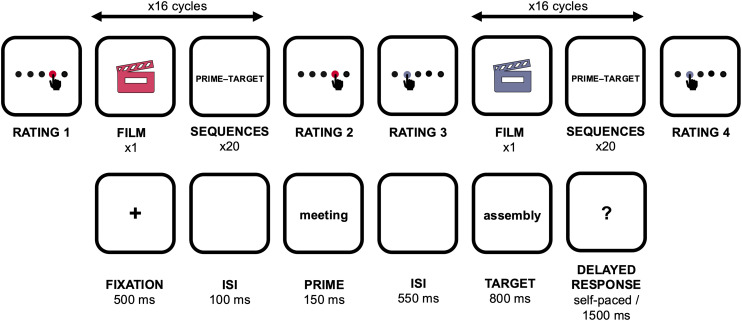
The experimental procedure and time sequence of stimulus presentation.

As part of the main experimental procedure, participants first watched a mood-inducing film and then made binary semantic relatedness judgements (i.e., decided if a given word pair was related or unrelated in meaning) about a sequence of 20 prime–target pairs. This procedure was repeated until all stimuli were presented, with continuous presentation of films allowing to sustain the elicited emotional states throughout each mood block. Each mood block included one Polish (L1) and one English (L2) block counterbalanced across participants, with 40 closely related, 40 weakly related, and 40 unrelated word pairs in each language block presented in a random order. Additional 40 unrelated filler prime–target pairs were added to the stimuli pool in each language block to balance the number of possible congruent (i.e., closely and weakly related) and incongruent (i.e., unrelated and filler) trials (*N*_*Total*_ = 640 pairs). No prime–target pair was repeated for any participant. Each target word appeared twice, but only in separate mood blocks and paired with a different prime each time. Fig 3 includes the time sequence of stimulus presentation. Response key assignment was counterbalanced across participants.

Note that participants made delayed responses (i.e., after 800 ms post stimulus onset) to avoid contamination of the potentials evoked by the target word with the potentials evoked by responses and response preparation. As the recorded response times were not temporally linked to the moment of decision-making, they may reflect more strategic processes rather than rapid, online mechanisms engaged during language processing. This also makes them less comparable to response times obtained in standard behavioural paradigms. Consequently, response times were used here as a broader measure of task performance and participant engagement rather than a fine-grained index of online processing dynamics (see S3 Appendix).

### 2.5. Data recording

The self-report background questionnaire and mood ratings were collected through SurveyMonkey (SurveyMonkey, Inc.). E-Prime 3.0 (Psychology Software Tools, Inc.) was used to present the stimuli and collect behavioural data (i.e., response times and response accuracy). The electrophysiological data were recorded at 512 Hz from 64 Ag/AgCl active electrodes placed at the International 10–20 system, with the BioSemi ActiveTwo amplifier and the ActiveView software (BioSemi B.V., Amsterdam, the Netherlands). The offset level was kept below 20mV.

### 2.6. Data analysis

#### 2.6.1. Self-report measures: Mood ratings.

The mood ratings analysis followed a 2 (Mood induction phase: Before vs. After mood induction) × 2 (Mood: Positive vs. Negative mood) within-subject design. Participants rated their current affective state using 7-point valence, arousal, and motivation scales (i.e., bipolar dimensions). To confirm that the mood manipulation was effective, we compared all mood ratings after relative to before mood induction separately in each mood condition as a planned comparison, expecting increased or comparable ratings in the positive mood condition and decreased mood ratings in the negative mood condition (see [3] for a review).

All statistical analyses were conducted in R [52]. The analysis of the mood ratings was performed using linear mixed-effect modelling (LMM) [53–55] using the *lme4* package [56]. A full random-effects structure was implemented in the initial maximal model, accounting for participant-specific intercepts and random slopes corresponding to fixed effects [54]. Model complexity was reduced to achieve parsimony via the application of principal component analysis (PCA) on the random-effects structure [57]. Specifically, we started with a maximal random-effects structure justified by the design and iteratively simplified it by removing variance components that contributed minimally to the overall model fit based on PCA diagnostics, following established recommendations [57].

We applied sum-to-zero contrasts to all predictor variables. Fixed effect estimates (*b*) and their significance (*p*-values) were derived using the Satterthwaite approximation via the *lmerTest* package [58]. Bonferroni-adjusted pairwise comparisons were computed via the *emmeans* package [59].

#### 2.6.2. Electrophysiological measures.

Electrophysiological data analyses were performed using EEGLAB [60] in Matlab R2024b (The MathWorks, Inc.). Continuous EEG data were filtered offline with a 0.1 Hz high-pass and 20 Hz low-pass using the Hamming windowed *sinc* FIR filter. Unsystematic artifacts in the continuous EEG data were manually identified and rejected. Noisy channels were identified using the *TrimOutlier* function and supplemented by visual inspection (*M*_*RejectedChannels*_ = 1.75, SD = 1.60, min = 0, max = 6). Next, the data were re-referenced to the global average across all electrodes. The continuous EEG data underwent Adaptive Mixture Independent Component Analysis (AMICA) [61]. The AMICA-derived components were classified using *ICLabel* [62] and those containing ocular artifacts were removed (*M*_*RejectedComponents*_ = 2.13, SD = 1.19, min = 0, max = 5). The cleaned data were then time-locked to word onset and epoched from −200 ms to 800 ms, followed by a baseline correction from −200 ms to 0 ms. Artifact rejection was refined by applying the *pop_jointprob* function, excluding epochs with extreme values exceeding 6 SDs at a single electrode and 3 SDs at all electrodes (*M*_*RejectedEpochs*_ = 11.43, SD = 4.78, min = 2, max = 28). Altogether, 4.11% of electrophysiological data were excluded during pre-processing.

The electrophysiological data analysis followed a 2 (Mood: Positive vs. Negative mood) × 3 (Word pair: Closely related vs. Weakly related vs. Unrelated pairs) within-subject design. Moreover, to account for potential changes in the strength of elicited mood effect over the course of the mood induction, we included Mood-induction progression (i.e., the number of mood‑inducing films watched so far within a block) as a continuous predictor in the models. This allowed us to assess whether mood effects on semantic processing remained stable or changed systematically as mood induction accumulated across the blocks.

In addition, we included a cosine-based semantic similarity measure as a continuous predictor in a separate model to capture graded variation in semantic relatedness beyond categorical condition labels. While this was not the primary focus of the analysis, it allowed us to explore whether more fine-grained differences in semantic similarity, contributed to the observed N400 responses, particularly in interaction with Mood and Word pair (see S4 Appendix).

ERP analysis was performed within a pre-defined N400 time window (300–500 ms) over three fronto-central (FC1, FCz, FC2), central (C1, Cz, C2), centro-parietal (CP1, CPz, CP2), and parietal (P1, Pz, P2) electrodes, in accordance with previous electrophysiological research on mood effects on language [36,40,63] and semantic priming [33]. As our hypothesis predicted interactive effects within and not between the two languages, separate analyses were conducted for Polish (L1) and English (L2) pairs [64,65]. Single trial-based mean ERP amplitudes were analysed with LMMs [53–55], similarly to mood ratings (see above).

We estimated the observed power to detect medium-to-large effects (Cohen’s *f* ≥ .25) for each reported model using the *smpsize_lmm* function from the *sjstats* R package [66]. All analyses assumed a significance level of *α* = .05, a sample of 32 participants, and 40 trials per condition. For each model, we reported the observed power and the intraclass correlation coefficient (ICC) in Table 6 and 7.

**Table 6:** Results of statistical analyses (statistics) of the N400 amplitudes evoked by both Polish (L1) and English (L2) pairs.

Effects	Closely related vs. Weakly related	Closely related vs. Unrelated	Weakly related vs. Unrelated
**Polish (L1) pairs: Fixed effect of Word pair**	*b* = −.44, SE = .12, *z* = −3.46, *p* = .002	*b* = −1.21, SE = .19, *z* = −6.41, *p* < .001	*b* = −.77, SE = .17, *z* = −4.42, *p* < .001
**Polish (L1) pairs: Mood × Word pair interaction (Positive mood)**	*b* = −.42, SE = .14, *z* = −3.04, *p* = .007	*b* = −1.21, SE = .20, *z* = −6.19, *p* < .001	*b* = −.79, SE = .18, *z* = −4.38, *p* < .001
**Polish (L1) pairs: Mood × Word pair interaction (Negative mood)**	*b* = −.47, SE = .14, *z* = −3.41, *p* = .020	*b* = −1.21, SE = .20, *z* = −6.21, *p* < .001	*b* = −.74, SE = .18, *z* = −4.12, *p* < .001
**English (L2): Fixed effect of Word pair**	*b* = −.60, SE = .05, *z* = −12.04, *p* < .001	*b* = −1.29, SE = .05, *z* = −26.22, *p* < .001	*b* = −.70, SE = .05, *z* = −14.16, *p* < .001

**Final models:** L1: Amplitude ~ Mood * Word pair * Mood-induction progression + (1 + Word pair | Participant) + (1 + Word Pair | Item) [observed power = .96, ICC = .058]; L2: Amplitude ~ Mood * Word pair * Mood-induction progression + (1 + Mood | Participant) + (1 | Item) [observed power = .97, ICC = .056] | CR – closely related pairs, WR – weakly related pairs, UR – unrelated pairs.

**Table 7:** Results of statistical analyses (EMMSs) of the N400 amplitudes evoked by both Polish (L1) and English (L2) pairs.

Effects	EMMs with 95% CIs
**Polish (L1) pairs: Fixed effect of Word pair**	CR: −.47 [−1.06, .12] WR: −1.24 [−1.79, −.68] UR: −1.68 [−2.22, −1.14]
**Polish (L1) pairs: Mood × Word pair interaction (Positive mood)**	CR: −.39 [−.98, −.20] WR: −1.18 [−1.73, −.62] UR: −1.60 [−2.14, −1.05]
**Polish (L1) pairs: Mood × Word pair interaction (Negative mood)**	CR: −.55 [−1.14, −.04] WR: −1.30 [−1.85, −.74] UR: −1.76 [−2.31, −1.22]
**English (L2): Fixed effect of Word pair**	CR: −.43 [−.94, .08] WR: −1.13 [−1.64, −.62] UR: −1.72 [−2.23, −1.21]

CR – closely related pairs, WR – weakly related pairs, UR – unrelated pairs, EMM – estimated marginal means, CI – confidence intervals.

## 3. Results

### 3.1. Self-report data: Mood ratings

**Fig 4:**
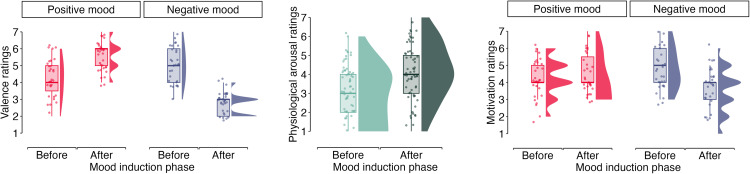
The panels provide a visual summary of changes in participants’ emotional and psychological states before and after mood induction, depicting valence (left), arousal (middle), and motivation (right) ratings.

The analysis of valence ratings showed fixed effects of Mood (*p* < .001) and Mood induction phase (*p* = .002), along with a Mood × Mood induction phase interaction. Planned comparisons showed an increase in valence ratings following mood induction in the positive mood condition (*p* < .001) and a decrease in the negative mood condition (*p* < .001; see Fig 4 and Table 4 and 5). The analysis of arousal ratings revealed a fixed effect of Mood induction phase, pointing to an overall increase in experienced physiological arousal following mood induction (*p* < .001; see Fig 4 and Table 4 and 5). The analysis of motivation ratings revealed a fixed effect of Mood induction phase (*p* = .003) and a Mood × Mood induction phase interaction. *Post-hoc* comparisons showed no difference in motivation following mood induction in the positive mood condition (*p* = .140) and a decrease in motivation in the negative mood condition (*p* < .001; see Fig 4 and Table 4 and 5).

**Table 4:** Results of statistical analyses of mood ratings (statistics).

Effects	Variable	Statistics
**Valence ratings: Fixed effects**	Mood	*b* = −.97, SE = .13, *z* = −7.33, *p* < .001
	Mood induction phase	*b* = .52, SE = .13, *z* = 3.91, *p* = .002
**Valence ratings: Mood** × **Mood induction phase interaction**	Positive Mood	*b* = −1.26, SE = .19, *z* = −6.74, *p* < .001
	Negative Mood	*b* = 2.29, SE = .19, *z* = 12.27, *p* <.001
**Arousal ratings: Fixed effect**	Mood induction phase	*b* = −.89, SE = .18, *z* = −5.03, *p* < .001
**Motivation ratings: Fixed effect**	Mood induction phase	*b* = .47, SE = .15, *z* = 3.08, *p* = .003
**Motivation ratings: Mood** × **Mood induction phase interaction**	Positive Mood	*b* = −.32, SE = .22, *z* = −1.50, *p* = .136
	Negative Mood	*b* = −1.26, SE = 1.26, *z* = 5.86, *p* < .001

**Final models:** Valence/arousal/motivation ratings ~ Mood * Mood induction phase + (1 | Participant).

**Table 5:** Results of statistical analyses of mood ratings (EMMs).

Effects	Variable	EMMs with 95% CIs
**Valence ratings: Fixed effects**	Mood	Positive mood: 4.92 [4.65, 5.19] Negative mood: 3.95 [3.68, 4.23]
	Mood induction phase	Before: 4.69 [4.42, 4.97] After: 4.18 [3.90, 4.45]
**Valence ratings: Mood** × **Mood induction phase interaction**	Positive Mood	Before: 4.29 [3.96, 4.62] After: 5.55 [5.22, 5.88]
	Negative Mood	Before: 5.10 [4.77, 5.43] After: 2.81 [2.48, 3.13]
**Arousal ratings: Fixed effect**	Mood induction phase	Before: 3.24 [2.80, 3.68] After: 4.13 [3.69, 4.57]
**Motivation ratings: Fixed effect**	Mood induction phase	Before: 4.52 [4.14, 4.89] After: 4.05 [3.67, 4.42]
**Motivation ratings: Mood** × **Mood induction phase interaction**	Positive Mood	Before: 4.26 [3.83, 4.15] After: 4.58 [4.15, 5.01]
	Negative Mood	Before: 4.77 [4.35, 5.20] After: 3.52 [3.09, 3.94]

EMM – estimated marginal means, CI – confidence intervals.

### 3.2. Electrophysiological data: N400 (300–500 ms)

#### 3.2.1. Polish (L1) prime–target pairs.

The analysis of the N400 responses elicited by Polish (L1) prime–target pairs showed a fixed effect of Word pair, such that unrelated pairs evoked higher N400 amplitudes than both weakly related (*p* = .002) and closely related pairs (*p* < .001). Moreover, weakly related pairs evoked higher amplitudes than closely related pairs (*p* < .001; see Table 6 and 7). There was also a fixed effect of Mood, whereby N400 amplitudes were overall attenuated in a positive compared to a negative mood (*b* = .15, SE = .04, *z* = 3.69, *p* = .002).

**Fig 5:**
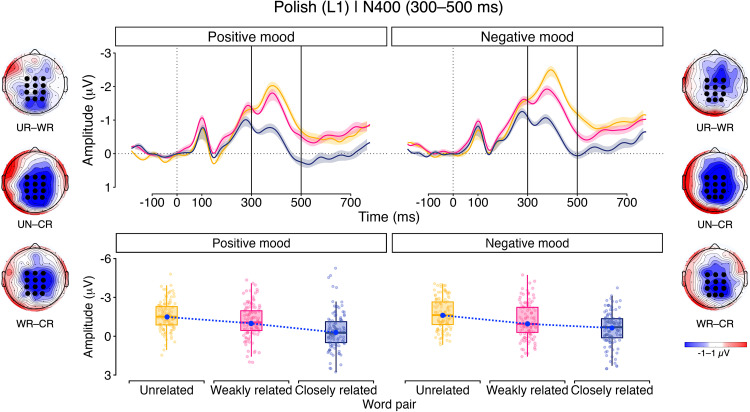
A visual representation of the Mood × Word pair interaction for Polish (L1) prime–target pairs in the N400 time window (300–500 ms). The shaded areas represent 95% confidence intervals. The waveforms (the top panel) represent brain potential variations over fronto-central (FC1, FCz, FC2), central (C1, Cz, C2), centro-parietal (CP1, CPz, CP2), and parietal (P1, Pz, P2) electrodes. The topographic maps (the outer left and right panels) represent differences in the N400 amplitudes between the respective word pair types in the 300–500 ms time window. The boxplots (the bottom panel) represent distribution of N400 responses to individual Polish (L1) prime–target pairs.

The analysis also showed a Mood × Word pair interaction. Besides the canonical graded effect of Word type in both mood conditions (see Table 6 and 7), *post-hoc* comparisons showed that Polish (L1) unrelated pairs (*b* = .17, SE = .07, *z* = 2.39, *p* = .017) as well as Polish (L1) closely related pairs (*b* = .16, SE = .07, *z* = 2.31, *p* = .021) evoked decreased N400 amplitudes in a positive compared to a negative mood, with no between-mood difference for weakly related pairs (*b* = .12, SE = .07, *z* = 1.69, *p* = .092; see Fig 5).

**Fig 6:**
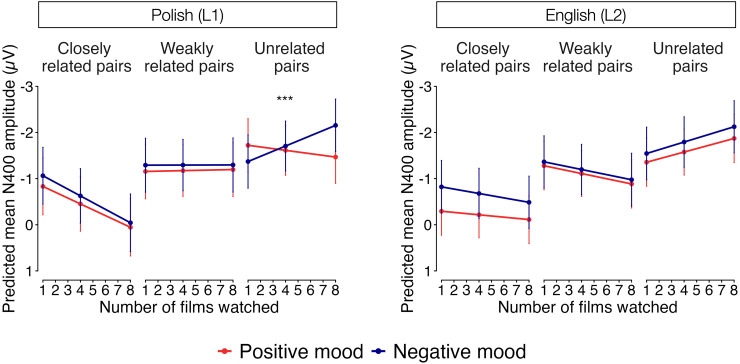
A visual representation of the Mood × Word pair × Mood-induction progression interaction for Polish (L1; left panel) and English (L2; right panel) prime–target pairs in the N400 time window (300–500 ms). Shown are model‑predicted mean N400 amplitudes as a function of mood‑induction progression (number of mood‑inducing films watched), separately for Mood and Word pair conditions. Lines represent linear mixed‑effects model predictions and error bars indicate 95% confidence intervals.

Finally, the analysis showed a Mood × Word pair × Mood-induction progression interaction. *Post-hoc* comparisons indicated that N400 mean amplitudes for Polish (L1) unrelated word pairs became progressively more pronounced as negative mood induction accumulated across the block (*b* = –.11, SE = .03, 95% CI [−.16, –.06]) and remained relatively stable as positive mood induction unfolded (*b* = .04, SE = .03, 95% CI [−.02,  .09]). The difference between these slopes was statistically significant (*b* = .15, SE = .04, *z* = 3.88, *p* < .001). For Polish (L1) weakly related word pairs, N400 amplitudes showed no reliable modulation as mood induction progressed in either a positive mood (*b* < –.01, SE = .03, 95% CI [−.06,  .05]) or a negative mood (*b* < –.01, SE = .03, 95% CI [−.05,  .05]), indicating relative temporal stability (*b* < –.01, SE = .04, *z* = –.13, *p* = .895). In contrast, Polish (L1) closely related word pairs exhibited a systematic modulation across the block, with N400 amplitudes becoming less negative as mood induction progressed in both positive (*b* = .13, SE = .03, 95% CI [.07,  .18]) and negative (*b* = .15, SE = .03, 95% CI [.09,  .20]) mood conditions, and no significant difference between the corresponding slopes across moods (*b* = –.02, SE = .04, *z* = –.52, *p* = .606; see Fig 6).

Pairwise comparisons of block‑related slopes within each mood condition further showed that, under a positive mood, Polish (L1) unrelated and weakly related word pairs did not differ in their temporal dynamics, whereas both unrelated and weakly related pairs differed from Polish (L1) closely related pairs (unrelated vs. weakly related: *b* = .04, SE = .04, *z* = 1.07, *p* = .285; unrelated vs. closely related: *b* = −.09, SE = .04, *z* = −2.34, *p* = .039; weakly vs. closely related: *b* = −.13, SE = .04, *z* = −3.43, *p* = .002). In contrast, under a negative mood, all three word-pair types differed significantly in their slopes, indicating a progressively graded semantic differentiation as negative mood induction accumulated (unrelated vs. weakly related: *b* = −.11, SE = .04, *z* = −2.92, *p* = .004; unrelated vs. closely related: *b* = −.26, SE = .04, *z* = −6.65, *p* < .001; weakly vs. closely related: *b* = −.15, SE = .04, *z* = −3.89, *p* < .001).

#### 3.2.2. English (L2) prime–target pairs.

**Fig 7:**
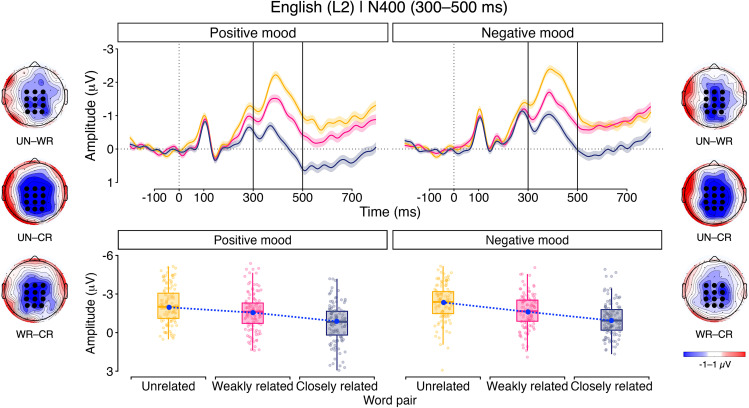
A visual representation of a fixed effect of Word pair split by both mood types for English (L2) prime–target pairs in the N400 time window (300–500 ms). The shaded areas represent 95% confidence intervals. The waveforms (the top panel) represent brain potential variations over fronto-central (FC1, FCz, FC2), central (C1, Cz, C2), centro-parietal (CP1, CPz, CP2), and parietal (P1, Pz, P2) electrodes. The topographic maps (the outer left and right panels) represent differences in the N400 amplitudes between the respective word pair types in the 300–500 ms time window. The boxplots (the bottom panel) represent distribution of N400 responses to individual English (L2) prime–target pairs.

The analysis of the N400 responses evoked by English (L2) prime–target pairs showed only a canonical graded fixed effect of Word pair, whereby unrelated pairs evoked higher N400 amplitudes than both weakly related (*p* < .001) and closely related pairs (*p* < .001). Moreover, weakly related pairs evoked higher amplitudes than closely related pairs (*p* < .001; see Fig 7 and Table 6 and 7).

## 4. Discussion

This electrophysiological study aimed to explore the neural dynamics underlying the relationship between mood and semantic processing in L1 and L2, in particular in the context of semantic relatedness. To this end, highly proficient female Polish–English bilinguals were induced into positive and negative moods with affectively potent animated films and performed semantic relatedness judgements on closely related words (e.g., *interpreter–translator*), weakly related words (e.g., *researcher–translator*), and unrelated words (e.g., *brake–translator*) in their L1 (Polish) and L2 (English). Overall, we observed patterns pointing to strong mood influences on the architecture of semantic memory in L1, with mood-independent effects in L2.

### 4.1. Facilitated activation within semantic memory under a positive mood in L1

In line with Hypothesis 1, we found strong electrophysiological evidence for facilitated spreading activation mechanisms within semantic memory in L1 under a positive compared to a negative mood, with unrelated prime–target pairs being particularly sensitive to positive mood influences. Specifically, N400 responses to L1 unrelated pairs were reduced in a positive compared to a negative mood. These findings accord well with those reported by Federmeier et al. [33] at a sentence level, showing decreased N400 responses to between-category violations (i.e., unexpected sentence endings from a different semantic category) in the positive mood condition compared to the baseline condition in female participants. In our study, unrelated pairs also elicited larger N400 amplitudes than weakly related pairs under a negative mood, while this difference also disappeared under a positive mood. Similarly, Federmeier et al. [33] reported no differences in the N400 amplitudes elicited by between-category and within-category (i.e., unexpected sentence endings from the same semantic category) violations. Together, such electrophysiological patterns indicate that a positive mood enhances spreading activation within semantic memory in L1, making distant concepts more accessible [27,33]. Put differently, such patterns indicate that a positive mood likely lowers the activation threshold for semantic nodes, allowing a wider set of concepts to become transiently activated, even when they share minimal associative or categorical overlap with the current input. Consequently, the semantic systems may temporarily treat remotely related or even unrelated concepts as accessible neighbours, thereby reducing the typical neural distinction between weak and unrelated semantic links under a positive mood.

Consistent with the *Affect-as-Information* framework [24,25], our findings support the view that a positive mood, unlike a negative mood, fosters assimilative processing. Put differently, a positive mood state promotes the integration of new information into pre-existing general knowledge structures, such as established schemas and associative networks, rather than subjecting information to a detailed scrutiny. Consequently, it results in more top-down processing which requires less cognitive effort than bottom-up analytical processing. Our results extend this account by demonstrating that, under a positive mood, activation within semantic memory can spread broadly, fostering a more flexible approach to semantic boundaries. Nevertheless, further research is needed to clarify the mechanisms underlying the facilitatory effects of a positive mood on activation within semantic memory, as available electrophysiological evidence also suggests that broadening of activation manifests in strengthening of associative links [34,35], rather than less categorical organisation of semantic knowledge [35].

Crucially, the analysis including mood‑induction progression further refines this interpretation by showing that the positive mood-driven facilitatory effect on L1 semantic processing was temporally stable, whereas the effects of a negative mood systematically intensified over the course of the block. Specifically, for unrelated word pairs, N400 amplitudes became progressively more pronounced as negative mood induction accumulated, while remaining relatively unchanged under a positive mood. This pattern suggests that, rather than reflecting a transient shift, a positive mood supports an early and sustained broadening of semantic activation, facilitating access even to distantly related concepts. In contrast, a negative mood may increasingly constrain semantic activation as the mood induction unfolds, effectively pushing the boundaries of accessible semantic space further apart, such that distant concepts become progressively harder to activate and are processed as more strongly mismatching.

Alternatively, our findings can also be interpreted in light of recent theoretical accounts that conceptualise mood as a dynamic modulator of predictive processing [67–69]. Indeed, we used a relatively long stimulus onset asynchrony of 700 ms, which might have encouraged more strategic, expectation-based mechanisms [70], potentially increasing the contribution of predictions and leading targets to be experienced as prediction errors. This is important for interpretation, as it suggests that the observed N400 effects may reflect not only automatic spreading activation, but also mood-related differences in how strongly predictions are formed and evaluated. For instance, Van de Cruys [67] proposed that a positive mood reduces the precision assigned to prediction errors, promoting flexible engagement and greater reliance on expectations. In contrast, a negative mood is argued to increase sensitivity to prediction errors, enhancing precision weighting and encouraging rapid model revision or even excessive updating. Therefore, the observed reduction of N400 amplitudes for unrelated pairs under a positive mood suggests that prediction errors were assigned lower precision, making mismatches less costly and allowing greater reliance on expectations. Conversely, larger N400 responses for unrelated pairs under a negative mood indicate heightened precision weighting of prediction errors, reinforcing strict semantic predictions and amplifying the cost of violations.

Finally, our analysis also revealed an unpredicted yet intriguing pattern: decreased N400 responses to closely related words in a positive compared to a negative mood. One possible explanation is linked to the nature of our stimuli, which were defined in terms of semantic relatedness but, in the case of closely related pairs, likely involved a higher density of inter-item connections. Consistent with this, an additional exploratory analysis using a cosine-based semantic similarity measure (see S4 Appendix) showed that, within closely related pairs, increasing similarity was associated with reduced N400 amplitudes in the positive mood condition, but not in the negative mood condition. Although such distributional measures primarily capture graded semantic similarity, they are often assumed to also reflect associative structure, given that co-occurrence patterns encode regularities in language use [47–49]. From this perspective, the observed pattern may tentatively point to an additional facilitation for more strongly associatively interconnected representations.

This interpretation is broadly consistent with earlier research on mood–semantics interactions, which has reported enhanced behavioural responses to highly relative to weakly associated prime–target pairs under a positive mood [30]. It may also relate to the notion of an *associative boost* [71], whereby semantic priming effects are amplified when items share both category membership and associative links [72,73]. Our findings extend this line of work by suggesting that, relative to a negative mood, a positive mood may enhance sensitivity to such graded relational strength among closely related concepts. At the same time, we stress that our design did not independently manipulate associative and categorical relations, and our similarity measure cannot unambiguously disentangle these dimensions. Future studies should therefore include orthogonal manipulations of semantic category membership and associative linkage to directly test whether mood selectively modulates associative, categorical, or combined forms of relational processing.

### 4.2. Mood-independent semantic processing in L2

Consistent with our Hypothesis 2, we observed a graded N400 pattern that remained unchanged across both mood conditions in L2, with the largest N400 amplitudes for L2 unrelated pairs, intermediate for L2 weakly related pairs, and the smallest for L2 closely related pairs. These patterns are in line with prior physiological [37] and electrophysiological evidence [36], pointing to mood-independent processing in L2. For instance, Naranowicz et al. [36] reported decreased N400 responses to meaningless sentences in L1 compared to L2 under a positive mood, with no between-language difference for any sentence type under a negative mood. Critically, a classic N400 meaningfulness effect (i.e., larger N400 responses to meaningless than meaningful sentences) was largely reduced only in L1 under a positive mood. Together, such electrophysiological evidence suggests that the broadened activation spread within semantic memory observed in L1 does not generalise to L2, which might imply that the L2 semantic network operates on more fixed structural constraints. At the same time, this interpretation should be treated with caution, as the absence of reliable mood effects in L2 may also reflect specific characteristics of the present task or participant profile. Indeed, previous behavioural and electrophysiological research revealed significant mood effects on L2 processing in contexts involving novel metaphors [40,74], gender stereotypes [75], and emotional words [38]. This indicates that mood-independent processing in L2 may be limited to mechanisms more directly related to semantic memory organisation, and that the interplay between mood and bilingual comprehension may remain dynamically modulated by various lexical, semantic, and social factors. Future research should therefore systematically examine the role of individual differences (e.g., L2 proficiency, affective sensitivity) as well as task-related factors (e.g., processing demands, stimulus type) in shaping mood effects on L2 semantic processing.

A possible explanation for the observed mood-independent effects lies in how operating in L2 influences bilinguals’ affective experiences. Growing physiological [37,76,77] and electrophysiological evidence [78–80] has pointed to attenuated emotional responding when operating in L2. This has also been linked with a phenomenon known as the *Foreign Language Effect* (FLE) [81], which refers to reduced emotional resonance and increased psychological distance when processing information in L2. Moreover, early evidence has suggested that L2 processing may automatically and implicitly down-regulate affective responses, which has been linked with increased cognitive control demands [82]. Thus, our findings extend previous FLE research by demonstrating that decreased affective resonance may also manifest in bilinguals through mood-independent effects on L2 comprehension [36].

Finally, as predicted, we observed a graded N400 effect in L2, with largest N400 responses to unrelated pairs, intermediate to weakly related pairs, and smallest to closely related pairs. Note that the same pattern was also observed in L1 under a negative mood. These findings align with extensive evidence suggesting that lexico-semantic processing in L1 and L2 may be comparable under certain conditions (see [22] for a review). Research has shown that one of the factors potentially minimising the difference between L1 and L2 processing in the N400 time window might be high L2 proficiency [16,23,83]. Based on Language History Questionnaire [43] as well as LexTALE [44], our bilingual participants were classified as highly proficient Polish (L1) learners of English (L2), who acquired English in instructional yet highly immersive learning context. Therefore, the expected similarities between L1 and L2 semantic processing in our study could be attributed to our participants’ high level of L2 proficiency.

### 4.3. Limitations

While the present study explored the interplay between mood and semantic processing in bilinguals, it focused on female participants with a very high L2 proficiency only. Consequently, our results might not be generalisable to other gender groups and a more heterogenous bilingual population. The deliberate choice of including only female participants intended to increase internal validity and minimise the influence of potential confounding variables. By limiting variation in participants’ demographic characteristics, we could restrict possible variabilities and isolate key factors for our study. The decision was also motivated by the prior evidence suggesting that mood effects on language processing tend to be more pronounced in females than males [35,38]. Future research should strive to include both male and female participants to increase the external validity of such investigations and more precisely explore psychophysiological interactions between mood and semantic memory in L1 and L2.

## 5. Conclusion

The present electrophysiological study explored the neural mechanisms behind the relationship between mood and semantic processing in L1 and L2, particularly in the context of semantic relatedness. The study offers novel electrophysiological evidence for facilitation of lexico-semantic mechanisms in L1 for semantically unrelated concepts (see also [35]). In line with the *Affect-as-Information* hypothesis [25], such patterns indicate that a positive mood, relative to a negative mood, promotes assimilative thinking and broadens spreading activation within semantic memory in L1 context, making distant concepts more accessible. We alternatively propose that such attenuation might be reflective of lowered precision assigned to prediction errors in a positive compared to a negative mood, which would make encountered mismatches less cognitively taxing, thereby enhancing the reliance on expectations under a positive mood [67–69].

Critically, in contrast to L1, lexico-semantic mechanisms in L2 remained unaffected by mood fluctuations [36], maintaining a more rigid pattern of semantic network navigation. This aligns with bilingual research pointing to decreased emotional responding when bilinguals operate in their L2 [37,80,81]. Altogether, these findings point to qualitative mood-dependent effects on semantic mechanisms in L1 and L2 and underscore the importance of considering mood as a dynamic factor shaping organisation of semantic memory along with predictive language comprehension, particularly in L1 contexts. They further suggest that models of bilingual semantic processing should integrate affective influences to better account for variability in cognitive flexibility and prediction mechanisms across languages.

## Supporting information

S1 Appendix Norming tests. (DOCX)

S2 Appendix Cosine-based semantic similarity. (DOCX)

S3 Appendix Behavioural data analysis. (DOCX)

S4 Appendix Cosine-based semantic similarity and N400 amplitude. (DOCX)
