# Supplementary material for: Positive mood enhances accessibility of unrelated concepts in the first language but not in the foreign language
Source: PLoS One. 2026 Aug 12;21(8):e0353990. doi: 10.1371/journal.pone.0353990 (PMC13465833; doi:10.1371/journal.pone.0353990)
Supplement: Supplementary file 1 [file pone.0353990.s001.docx]

**S1 Appendix: Norming tests**

**Materials.** The stimuli used in in the present study included Polish and English nouns controlled for frequency, concreteness, length (i.e., the number of characters), valence, physiological arousal, and age of acquisition (see **S1 Table 1** for details). Having excluded Polish–English cognates, polysemous words, as well as interlingual homographs and homophones [1] we chose 140 target words in Polish (native language; L1) and 155 target words in English (foreign language; L2). Each target word was paired with three primes and assigned to three unique semantic relatedness categories: closely related, weakly related, and unrelated word pairs.

**S1 Table 1.** Means (with 95% confidence intervals in square brackets and ranges in round brackets) for the lexico-semantic characteristics of Polish and English words.

| **Characteristic** | **Language** | **Targets** | **Closely related primes** | **Weakly related primes** | **Unrelated**  **primes** |
| --- | --- | --- | --- | --- | --- |
| **Frequency^1^** | Polish | 3.30 [3.24, 3.37], (1.53–5.33) | 3.42 [3.35, 3.49], (1.68–4.98) | 3.33 [3.26, 3.40], (1.98–5.20) | 3.19 [3.13, 3.26], (2.01–4.71) |
|  | English | 3.91 [3.85, 3.98], (2.30–5.30) | 3.95 [3.88, 4.33], (2.10–5.90) | 3.78 [3.72, 3.85], (2.30–5.20) | 4.06 [4.01, 4.12], (2.90–5.20) |
| **Concreteness^2^** | Polish | 2.09 [2.04, 2.13], (1.44–3.82) | 2.15 [2.09, 2.22], (1.50–5.70) | 2.13 [2.06, 2.21], (1.50–6.00) | 2.18 [2.13, 2.23], (1.40–3.50) |
|  | English | 1.99 [1.91, 2.07], (1.00–4.30) | 2.16 [2.04, 2.29], (1.00–5.40) | 2.36 [2.22, 2.51], (1.00–7.50) | 1.49 [1.43, 1.54], (1.00–3.70) |
| **Length** | Polish | 5.70 [5.54, 5.86], (3.00–10.00) | 5.81 [5.64, 5.98], (3.00–12.00) | 5.94 [5.76, 6.13], (3.00–10.00) | 6.21 [6.03, 6.39], (3.00–11.00) |
|  | English | 5.75 [5.55, 5.95], (3.00–10.00) | 5.78 [5.60, 5.97], (3.00–12.00) | 6.32 [6.09, 6.55], (3.00–14.00) | 5.28 [5.12, 5.45], (3.00–11.00) |
| **Valence^3^** | Polish | 5.20 [5.16, 5.25], (3.94–6.14) | 5.28 [5.20, 5.35], (2.50–7.20) | 5.48 [5.40, 5.55], (3.60–7.70) | 5.24 [5.19, 5.29], (3.50–6.20) |
|  | English | 5.25 [5.18, 5.32], (2.90–7.50) | 5.44 [5.35, 5.54], (2.70–7.50) | 5.43 [5.34, 5.53], (2.60–7.70) | 5.66 [5.56, 5.75], (3.10–7.60) |
| **Arousal^3^** | Polish | 3.44 [3.39, 3.49] (2.70–5.02) | 3.47 [3.41, 3.53], (2.60–5.40) | 3.49 [3.43, 3.55], (2.50–4.80) | 3.47 [3.42, 3.52], (2.60–5.20) |
|  | English | 3.85 [3.77, 3.93], (2.30–6.30) | 3.92 [3.84, 4.00], (2.40–6.40) | 3.77 [3.69, 3.85], (1.80–5.80) | 3.77 [3.69, 3.86], (2.30–6.80) |
| **Age of acquisition^3^** | Polish | 8.13 [7.98, 8.28], (5.00–12.00) | 8.03 [7.88, 8.17], (5.80-12.20) | 7.94 [7.79, 8.09], (4.90-11.60) | 8.27 [8.11, 8.42], (5.80–12.60) |
|  | English | 7.26 [7.03, 7.49], (3.00–12.80) | 7.28 [7.03, 7.53], (5.80–12.20) | 7.80 [7.55, 8.04], (2.50–13.90) | 5.92 [5.74, 6.10], (2.90–12.40) |

^1^Based on SUBTLEX-UK [2] and SUBTLEX-PL [3] (the Zipf scale): 1 – the lowest frequency, 7 – the highest frequency. ^2^ Based on Imbir [4] for Polish words and Brysbaert et al. [5] for English words: 1 – abstract abstract word, 7 – concrete word. ^3^Based on Imbir [4] for Polish words and Warriner et al. [6] for English words: 1 – strongly negative/unarousing word, 7 – strongly positive/arousing word

**Raters.** The word pairs were originally rated by 90 highly proficient L1-dominant Polish–English bilinguals (L1–L2), 7 of whom were excluded from the analyses due strong response biases (e.g., choosing only the middle score throughout). Consequently, 83 participants aged 19–29 (*M_Age_* = 22.06, 95% CI [21.53, 22.59]; 62 women, 14 men, 1 transgender man, and 6 non-binary individuals) completed the surveys. Self-declared proficiency ranged from C1 to C2 in Polish (L1) and B2 to C2 in English (L2). Raters were recruited from the population of students and graduates of the Faculty of English, Adam Mickiewicz University, Poznań (AMU). They received credit points for participation.

**Procedure.** Stimuli included 885 prime–target pairs that divided into 6 separate online surveys, with 147–148 pairs in each. Each survey included two language blocks (one Polish and one English block), whose presentation was counterbalanced across participants. The word pairs in each survey were presented in a pseudorandom order. Each prime–target pair was rated by 20 raters. While each rater completed a single survey once, they were allowed to complete all of them. They were asked to rate the prime and target in each pair in terms of their relatedness in meaning on a 7–point Likert scale (1 – *very unrelated words/bardzo niepowiązane słowa*, 7 – *very related words/bardzo powiązane słowa*). Ratings were collected online using Microsoft Forms [7]. The completion of a single survey took about 10 minutes. Based on the obtained ratings, we selected 120 Polish and 120 English prime–target pairs that best fitted their respective categories and allowed us to match the items across the two languages (see below).

**Data analysis.** All analyses were performed in R [8]. The relatedness ratings were analysed using linear mixed-effects models (LMMs) with the *lme4* package for R. Interclass correlation coefficients (ICC) were computed using a two-way mixed-effect model based on the mean of ratings of multiple participants to assess inter-rater reliability. Additionally, Cronbach’s alpha coefficients were calculated to evaluate the internal consistency of the relatedness ratings across all surveys.

**Reliability.** ICCs indicated good inter-rater reliability in all six surveys (*M_ICC_* = .81). Furthermore, ratings in all six surveys showed excellent internal consistency (*M_α_* = .94).

**Results.** The analysis of semantic relatedness ratings revealed a fixed effect of Word pair, whereby closely related word pairs were rated higher on the semantic relatedness scale relative to both weakly related (*p* < .001) and unrelated word pairs (*p* < .001). Then, weakly related word pairs were rated higher relative to unrelated word pairs (*p* < .001). There was also a Language × Word pair interaction. *Post-hoc* comparisons revealed only a marginally significant trend towards higher semantic relatedness ratings for unrelated pairs in English compared to Polish (*p* = .06), yet there were no differences in the semantic relatedness ratings between the two languages in closely related *(p* = .84) and weakly related (*p* = .39) pairs (see **S1 Table 2**).

**S1 Table 2.** Results of statistical analyses of the semantic relatedness ratings.

| **Fixed effect of Word pair** | | | |
| --- | --- | --- | --- |
| **CR vs. WR** | **CR vs. UR** | **WR vs. UR** | **EMMs with 95% CIs** |
| *b* = 1.42, SE = .05,  *z* = 27.40, *p* < .001 | *b* = 4.85, SE = .06,  *z* = 81.01, *p* < .001 | *b* = 3.42, SE = .06,  *z* = 45.83, *p* < .001 | CR: 5.94 [5.82, 6.05]  WR: 4.51 [4.36, 4.67]  UR: 1.09 [1.04, 1.14] |
| **Language × Word pair interaction** | | | |
| **CR in Polish vs.**  **CR in English** | **WR in Polish vs.**  **WR in English** | **UR in Polish vs.**  **UR in English** | **EMMs with 95% CIs** |
| *b* = –.01, SE = .05,  *z* = –.21, *p* = .840 | *b* = .04, SE = .05,  *z* = .86, *p* = .390 | *b* = –.09, SE = .05,  *z* = –1.87, *p* = .060 | Polish CR: 5.86 [5.86, 6.07]  English CR: 5.87 [5.87, 6.08]  Polish WR: 4.43 [4.43, 4.64]  English WR: 4.39 [4.39, 4.60]  Polish UR: 1.03 [.93, 1.14]  English UR: 1.12 [1.02, 1.22] |

**Final model:** Mean rating ~ Language * Word pair + (1 + Language + Word pair | Participant) + (1 | Target) | CR – closely related pairs, WR – weakly related pairs, UR – unrelated pairs, EMM – estimated marginal mean, CI – confidence intervals

**References**

**1.** Kroll JF, Dussias PE, Bogulski CA, Kroff JRV. Juggling Two Languages in One Mind. Psychology of Learning and Motivation. Elsevier; 2012. pp. 229–262. doi:10.1016/B978-0-12-394393-4.00007-8

**2.** Van Heuven WJB, Mandera P, Keuleers E, Brysbaert M. Subtlex-UK: A New and Improved Word Frequency Database for British English. Quarterly Journal of Experimental Psychology. 2014;67: 1176–1190. doi:10.1080/17470218.2013.850521

**3.** Mandera P, Keuleers E, Wodniecka Z, Brysbaert M. Subtlex-pl: subtitle-based word frequency estimates for Polish. Behav Res. 2015;47: 471–483. doi:10.3758/s13428-014-0489-4

**4.** Imbir KK. Affective Norms for 4900 Polish Words Reload (ANPW_R): Assessments for Valence, Arousal, Dominance, Origin, Significance, Concreteness, Imageability and, Age of Acquisition. Front Psychol. 2016;7: 1081. doi:10.3389/fpsyg.2016.01081

**5.** Brysbaert M, Warriner AB, Kuperman V. Concreteness ratings for 40 thousand generally known English word lemmas. Behav Res. 2014;46: 904–911. doi:10.3758/s13428-013-0403-5

**6.** Warriner AB, Kuperman V, Brysbaert M. Norms of valence, arousal, and dominance for 13,915 English lemmas. Behav Res. 2013;45: 1191–1207. doi:10.3758/s13428-012-0314-x

**7.** Microsoft Corporation. Microsoft Forms - Free tool to create online surveys, forms, polls, and quizzes. [cited 10 Dec 2025]. Available: <https://forms.office.com/?showstatic=1>
