# Supplementary material for: Positive mood enhances accessibility of unrelated concepts in the first language but not in the foreign language
Source: PLoS One. 2026 Aug 12;21(8):e0353990. doi: 10.1371/journal.pone.0353990 (PMC13465833; doi:10.1371/journal.pone.0353990)
Supplement: Supplementary file 2 [file pone.0353990.s002.docx]

**S2 Appendix: Cosine-based semantic similarity**

**Results.** The analysis of cosine-based semantic similarity revealed a fixed effect of Word pair, whereby closely related word pairs had higher semantic similarity values relative to both weakly related (*p* < .001) and unrelated word pairs (*p* < .001). Then, weakly related word pairs had higher semantic similarity values relative to unrelated word pairs (*p* < .001). There was also a Language × Word pair interaction. *Post-hoc* comparisons revealed that closely related pairs had higher semantic similarity values in Polish than English (*p* = .006) while weakly related and unrelated pairs had higher semantic similarity values in Polish than English (*p* < .044; see **S2 Table 1**). Notably, although these differences were statistically significant, their absolute magnitude was relatively small (between .03 and .05) given that cosine similarity values are bounded between −1 and 1.

**S2 Table 1.** Results of statistical analyses of cosine-based semantic similarity.

| **Fixed effect of Word pair** | | | |
| --- | --- | --- | --- |
| **CR vs. WR** | **CR vs. UR** | **WR vs. UR** | **EMMs with 95% CIs** |
| *b* = –.15, SE < .01,  *z* = –15.61, *p* < .001 | *b* = –.37, SE < .01,  *z* = –39.14, *p* < .001 | *b* = –.23, SE < .01,  *z* = –23.52, *p* < .001 | CR: .49 [.48, .51]  WR: .35 [.33, .36]  UR: .12 [.11, .13] |
| **Language × Word pair interaction** | | | |
| **CR in Polish vs.**  **CR in English** | **WR in Polish vs.**  **WR in English** | **UR in Polish vs.**  **UR in English** | **EMMs with 95% CIs** |
| *b* = –.04, SE = .01,  *z* = –2.78, *p* = .006 | *b* = .03, SE = .01,  *z* = 2.02, *p* = .044 | *b* = .05, SE = .01,  *z* = 3.39, *p* < .001 | Polish CR: .47 [.46, .49]  English CR: .51 [.49, .53]  Polish WR: .36 [.34, .38]  English WR: .33 [.31, .35]  Polish UR: .14 [.12, .16]  English UR: .10 [.08, .12] |

**Final model:** Mean rating ~ Language * Word pair + (1 | Target) | CR – closely related pairs, WR – weakly related pairs, UR – unrelated pairs, EMM – estimated marginal mean, CI – confidence intervals
