# Supplementary material for: Positive mood enhances accessibility of unrelated concepts in the first language but not in the foreign language
Source: PLoS One. 2026 Aug 12;21(8):e0353990. doi: 10.1371/journal.pone.0353990 (PMC13465833; doi:10.1371/journal.pone.0353990)
Supplement: Supplementary file 3 [file pone.0353990.s003.docx]

**S3 Appendix: Behavioural data analysis**

**Data analysis.** All analyses of behavioural data were performed in R [1]. Response times data were analysed using linear mixed-effects models and response accuracy data with generalised linear- generalized (logistic) linear mixed-effects model [2, 3, 4, 5]. The analyses followed

**Response accuracy: Polish (L1).** The analysis of response accuracy for Polish (L1) pairs revealed a fixed effect of Word pair, such that, in Polish (L1), unrelated pairs were responded to with higher accuracy than both closely related (*p* = .019) and weakly related pairs (*p* < .001). Moreover, closely related pairs were responded to with higher accuracy than weakly related pairs (*p* < .001; see **S3 Fig 1**; **S3 Table 1**).

**Response accuracy: English (L2).** The analysis of response accuracy for English (L2) pairs revealed a fixed effect of Word pair, whereby, in English (L2), unrelated pairs were responded to with higher accuracy than both closely related (*p* < .001) and weakly related pairs (*p* < .001). Moreover, closely related pairs were responded to with higher accuracy than weakly related pairs (*p* < .001; see **S3 Fig 2; S3 Table 1**).

**S3 Fig 1.** Results of response accuracy analysis for Polish (L1) and English (L2) pairs.

**Response times: Polish (L1).** The analysis of response times for Polish (L1) pairs revealed a fixed effect of Word pair, such that, in Polish (L1), weakly related pairs were responded to slower than both closely related pairs (*p* < .001) and unrelated pairs (*p* < .001), with no difference between closely related and unrelated pairs (*p* = 1.00; see **S3 Fig 1**; **S3 Table 1**).

**Response times: English (L2).** The analysis of response times for English (L2) pairs revealed a fixed effect of Word pair, whereby, in English (L2), weakly related pairs were responded to slower than both closely related pairs (*p* < .001) and unrelated pairs (*p* < .001), with no difference between closely related and unrelated pairs (*p* = 1.00; see **S3 Fig 2**; **S3 Table 1**).

**S3 Fig 2.** Results of response times analysis for Polish (L1) and English (L2) pairs.

**S3 Table 1.** Results of statistical analyses of behavioural data.

| **Closely related**  **vs. Weakly related** | **Closely related**  **vs. Unrelated** | **Weakly related**  **vs. Unrelated** | **EMMs with 95% CIs** |
| --- | --- | --- | --- |
| **Response accuracy \| Polish (L1) pairs: Fixed effect of Word pair** | | | |
| *b* = –2.80, SE = .33,  *z* = –8.56, *p* < .001 | *b* = 3.10, SE = 1.14,  *z* = 2.72, *p* = .019 | *b* = 5.89, SE = 1.12,  *z* = 5.27, *p* < .001 | CR: 97.18% [96.54, 97.81]  WR: 73.68% [71.99, 75.36]  UR: 99.16% [98.81, 99.51] |
| **Response accuracy \| English (L2) pairs: Fixed effect of Word pair** | | | |
| *b* = –2.-4, SE = .25,  *z* = –8.03, *p* < .001 | *b* = 1.72, SE = .43,  *z* = 3.96, *p* < .001 | *b* = 3.76, SE = .44,  *z* = 8.58, *p* < .001 | CR: 88.78% [87.57, 89.99]  WR: 68.05% [66.27, 69.84]  UR: 98.08% [97.56, 98.61] |
| **Response times \| Polish (L1) pairs: Fixed effect of Word pair** | | | |
| *b* = .20, SE = .03,  *z* = 8.66, *p* < .001 | *b* = .02, SE = .03,  *z* = .89, *p* = 1.00 | *b* = –.17, SE = .03,  *z* = –5.78, *p* < .001 | CR: 272.92 ms [266.27, 279.57]  WR: 329.54 ms [321.52, 337.55]  UR: 280.07 ms [273.32, 286.81] |
| **Response times \| English (L2) pairs: Fixed effect of Word pair** | | | |
| *b* = .17, SE = .02,  *z* = 8.79, *p* < .001 | *b* = .01, SE = .02,  *z* = .50, *p* = 1.00 | *b* = –.16, SE = .02,  *z* = –7.75, *p* < .001 | CR: 300.23 ms [282.41, 308.05]  WR: 351.66 ms [342.78, 360.55]  UR: 308.02 ms [299.81, 316.23] |

**Final models:** Response accuracy –– L1: Accuracy ~ Mood * Word pair + (1 + Word pair | Participant) + (1 + Word pair | Item); L2: Accuracy ~ Mood * Word pair + (1 + Mood | Participant) + (1 + Word pair | Item) | Response times –– L1: Response times ~ Mood * Word pair + (1 + Word pair | Participant) + (1 + Word pair | Item); L2: Response times ~ Mood * Word pair + (1 + Mood + Word pair | Participant) + (1 + Word pair | Item) | EMM – estimated marginal mean, CI – confidence intervals

**References**

**1.** R: The R Project for Statistical Computing. [cited 10 Dec 2025]. Available: <https://www.r-project.org/>

**2.** Baayen RH, Davidson DJ, Bates DM. Mixed-effects modeling with crossed random effects for subjects and items. Journal of Memory and Language. 2008;59: 390–412. doi:10.1016/j.jml.2007.12.005

**3.** Barr DJ. Random effects structure for testing interactions in linear mixed-effects models. Front Psychol. 2013;4. doi:10.3389/fpsyg.2013.00328

**4.** Barr DJ, Levy R, Scheepers C, Tily HJ. Random effects structure for confirmatory hypothesis testing: Keep it maximal. Journal of Memory and Language. 2013;68: 255–278. doi:10.1016/j.jml.2012.11.001

**5.** Jaeger TF. Categorical data analysis: Away from ANOVAs (transformation or not) and towards logit mixed models. Journal of Memory and Language. 2008;59: 434–446. doi:10.1016/j.jml.2007.11.007
