# Supplementary material for: Positive mood enhances accessibility of unrelated concepts in the first language but not in the foreign language
Source: PLoS One. 2026 Aug 12;21(8):e0353990. doi: 10.1371/journal.pone.0353990 (PMC13465833; doi:10.1371/journal.pone.0353990)
Supplement: Supplementary file 4 [file pone.0353990.s004.docx]

**S4 Appendix: Cosine-based semantic similarity and N400 amplitude**

**Results: Polish (L1).** The analysis of the N400 responses (300–500 ms) to Polish (L1) pairs revealed a Mood × Word pair × Semantic similarity interaction. *Post-hoc* trend estimates indicated that, for Polish (L1) closely related word pairs, N400 mean amplitudes became less negative with increasing semantic similarity in the positive mood condition (*b* = 2.66, SE = 0.84, 95% CI [1.01, 4.31]), with no effect in the negative mood condition (*b* = 0.15, SE = 0.83, 95% CI [−1.47, 1.77]). For weakly related pairs, N400 amplitudes showed no reliable modulation as a function of semantic similarity in either a positive mood (*b* = 0.31, SE = 0.85, 95% CI [−1.35, 1.97]) or a negative mood (*b* = 0.28, SE = 0.82, 95% CI [−1.34, 1.89]). Similarly, for unrelated pairs, the effect of semantic similarity was not reliable in either a positive (*b* = −0.78, SE = 1.16, 95% CI [−3.06, 1.50]) or a negative mood (*b* = 0.96, SE = 1.16, 95% CI [−1.31, 3.23]).

Pairwise comparisons of semantic similarity slopes within each mood condition showed that, under positive mood, the slope for closely related pairs differed from that for unrelated pairs (*b* = −3.44, SE = 1.44, *z* = −2.39, *p* = .050), while differences between unrelated and weakly related pairs (*b* = −1.09, SE = 1.43, *z* = −0.76, *p* = .447) and between weakly and closely related pairs (*b* = −2.35, SE = 1.19, *z* = −1.97, *p* = .098) did not reach significance. In contrast, under a negative mood, no significant differences between word-pair types were observed (all *p*s = 1.00), indicating comparable and overall weak modulation by semantic similarity across conditions (see **S4** F**ig 1** below).

**Results: English (L2).** The analysis of the N400 responses (300–500 ms) to English (L2) pairs revealed no significant fixed effect of or interactions with Semantic similarity.

**S4 Fig 1.** A visual representation of the Mood × Word pair × Semantic similarity interaction for Polish (L1) prime–target pairs in the N400 time window (300–500 ms). Shown are model‑predicted mean N400 amplitudes as a function of cosine-based semantic similarity, separately for mood and word pair conditions. Lines represent linear mixed‑effects model predictions and error bars indicate 95% confidence intervals.
